# Consensus on Care Competencies for Community Citizens in Japan: A Modified Delphi Study

**DOI:** 10.3390/ijerph22121774

**Published:** 2025-11-24

**Authors:** Manami Takaoka, Ayumi Igarashi, Taisuke Yasaka, Yuka Sumikawa, Kyoko Yoshioka-Maeda, Chikako Honda, Hiroshige Matsumoto, Haruna Kugai, Asako Futami, Noriko Yamamoto-Mitani

**Affiliations:** 1 Department of Advanced Gerontological Nursing, Graduate School of Nursing, School of Nursing, Chiba University, Chiba 2600856, Japan; 2 Department of Nursing Data Science, Graduate School of Medicine, The University of Tokyo, Tokyo 1130033, Japan; 3 Department of Gerontological Home Care and Long-Term Care Nursing, Graduate School of Medicine, The University of Tokyo, Tokyo 1130033, Japannoriko-tky@g.ecc.u-tokyo.ac.jp (N.Y.-M.); 4 Department of Community Health Nursing/Public Health Nursing, Graduate School of Medicine, The University of Tokyo, Tokyo 1130033, Japanhchika-tky@g.ecc.u-tokyo.ac.jp (C.H.); hiroshige-tky@g.ecc.u-tokyo.ac.jp (H.M.)

**Keywords:** care competency, Delphi study, nursing, citizen, community symbiotic society

## Abstract

Japan’s rapidly aging population necessitates new approaches that enable citizens to actively participate in caring for themselves and others. However, a comprehensive framework defining the specific competencies needed for this critical community role has not yet been established. This study, therefore, aimed to define the novel concept of “care competency” and establish a consensus on its comprehensive component list for community citizens. We defined care competencies and developed a list using a modified Delphi technique (RAND/University of California, Los Angeles) involving 10 nursing researchers. Items were adapted from Japan’s Model Core Curriculum for Nursing Education, and a total of 528 items were evaluated and refined. In this study, care competency was defined as the complex ability to acquire and utilize knowledge and skills, based on evidence and intentional choices, to maintain the well-being of oneself, loved ones, and people in the community. The Delphi process identified 151 care competency items. This study thereby presents a novel framework that provides a foundation for developing globally applicable educational programs to foster mutual support and effective caregiving.

## 1. Introduction

Community-based care models are crucial in rapidly aging societies globally, and the need for integrated care approaches is vital for safeguarding the well-being of citizens in our communities [1,2]. Japan has one of the fastest-aging populations in the world, and with the proportion of people aged 65 and over projected to reach 38.7% by 2070 [3], community-based care models are indispensable. In these models, citizens—including family members and neighbors—are encouraged to assume an important role in providing care [4]. To effectively fulfill their roles in a community-based, inclusive society, each community member is expected to acquire the necessary knowledge and skills related to caring for themselves and others through education and training.

Effective education and training require a well-grounded theoretical basis and a conceptual model. Existing concepts related to the acquisition of knowledge and skills in caring for themselves and others include health literacy [5,6,7], community literacy [8], and care literacy [9]. Health literacy, a concept related to the acquisition of knowledge and skills in caring for oneself and others, refers to the degree to which individuals can obtain, process, and understand the basic health information and services needed to make appropriate health decisions [5,6,7]. However, a critical gap in existing frameworks is that they focus mainly on understanding and lack the practical dimensions of caregiving, such as empathic attitudes, role readiness, and contextual decision-making—skills that are crucial for non-professionals. Consequently, they do not fully capture the complex ability to integrate and apply knowledge, attitudes, and practical skills in real-world scenarios. To address this critical gap and enhance training programs, we propose the novel and comprehensive concept of “care competency.”

Care competency is defined as the complex ability to acquire and utilize knowledge and skills, based on evidence and intentional choices, to maintain the well-being of oneself, loved ones, and the community. We labeled this concept “care competency” because it includes knowledge and literacy as well as the skills and ability to implement them in practice. Currently, programs are being implemented to equip various citizens with the knowledge and skills required for caregiving [10,11,12]. In Japan, numerous specific “care training programs” have been established, such as dementia supporter [13] and mental health peer supporter [14] training programs. While many programs focus on specific diseases or caregiving techniques, none are based on a comprehensive competency framework. Therefore, comprehensive programs based on a framework that addresses the necessary knowledge, attitudes, and skills required to provide and receive care are needed.

Thus, this study aimed to clarify the concept and components of care competencies based on expert consensus using the Delphi method, a structured technique that allows for the systematic evaluation and selection of conceptual items.

## 2. Materials and Methods

### 2.1. Study Design

This study comprised two phases as follows: (1) defining care competencies, and (2) conducting two expert rating rounds using the RAND/University of California, Los Angeles (UCLA) method, an approach jointly developed by the RAND Corporation and UCLA [15]. The RAND/UCLA (modified Delphi) method was selected as the most appropriate consensus group method as it allows for beginning with selected items grounded in previous work [16].

We used the Model Core Curriculum for Nursing Education in Japan [17] as the initial definition of the competencies required for community citizens. We then used a modified Delphi approach (the RAND/UCLA method) over two rating rounds with stakeholders to assess and select items from the Model Core Curriculum for community citizens who are non-healthcare professionals. The RAND/UCLA method provides a systematic approach to evaluating each item by combining quantitative ratings and qualitative discussions. Each item was independently rated on a 9-point scale for its importance, followed by statistical analysis of the median and interquartile ranges to identify the level of agreement. Where there was disagreement over individual items, these were reviewed and discussed in panel meetings to reach a consensus, ensuring that the final selection reflected both empirical assessment and expert judgment. In setting the criteria for agreement and disagreement, we referred to the RAND/UCLA Appropriateness Method User’s Manual (pp. 59–63), which defines the thresholds for panel sizes of 8–9 members. Although our panel consisted of 10 members and did not exactly match the 9-member model, we adopted comparable criteria to maintain consistency with the RAND/UCLA framework and ensure a structured, evidence-based consensus process [15].

### 2.2. Panelists

The panelists in this study comprised 10 nursing researchers, all holding nursing credentials in Japan. This diverse group of nursing professionals had various areas of expertise, ensuring a wide range of perspectives on care competencies. As the study used the Model Core Curriculum for Nursing Education in Japan as its starting point, the selection criteria specifically required that all panelists hold a registered nurse license, and nine members also possessed a Public Health Nurse qualification. Together, the panelists’ expertise covered all competency domains, including community, acute care, geriatric, home visiting, public health, and pediatric nursing. They were recruited through an opportunistic call for research collaboration, with each individual volunteering to participate. A key selection criterion was prior working experience as a nurse or public health nurse in either clinical (hospital) or administrative settings.

### 2.3. Defining Care Competencies and List Development

The process commenced with an in-depth discussion among the researchers, who collaboratively reviewed the existing literature to establish a definition of care competency. We examined textbooks from elementary, junior high, and high schools, alongside the citizen education programs implemented by companies and local governments, to determine how they align with the Model Core Curriculum of Nursing Education. This analysis facilitated the identification of trends within the current content of education and training. This step was especially crucial for clearly understanding the skills, knowledge, and attitudes essential for community members who are non-healthcare professionals.

The Model Core Curriculum for Nursing Education in Japan [15] outlines the foundational knowledge and skills that nursing professionals are expected to acquire during their basic education. We selected this rigorous professional curriculum because it provides a comprehensive structure encompassing the necessary knowledge, attitudes, and practical skills essential for safeguarding health and well-being, as compared with existing non-professional training programs that usually lack a comprehensive theoretical basis and the integration of attitudes and practical skills. Given that the nursing profession’s training program curriculum includes essential elements for comprehensive professional development, it serves as an appropriate and robust basis for systematically developing care competencies tailored to community members who are non-healthcare professionals. We pooled 528 items from the curriculum for further analysis.

### 2.4. Delphi Surveys Using the RAND/UCLA Method

We conducted a two-round rating process between November 2022 and June 2023. In each round, the core elements of each item were rated on a 9-point Likert scale from 1 (“not essential”) to 9 (“essential”) [18], and the results were analyzed to identify any disagreements. Statistical analyses were conducted by two researchers who were not part of the 10-person expert panel (MT and TY) to ensure the integrity of the Delphi process. Their responsibilities included calculating the median ratings, interquartile ranges, and levels of agreement for each item, as well as moderating the panel meetings. Access to each other’s ratings was restricted throughout the rating period to minimize potential bias among the panelists. Each panelist then independently reviewed the competencies and assigned scores. At this phase, panelists were allowed to view aggregated scoring data and statistical information—such as median ratings and the number of raters for each score—during the panel meetings. Although the panelists’ scores were disclosed, the identities of the individuals who provided each score remained anonymous. This approach ensured that the ratings were not influenced by the status or perceived authority of any individual rater.

#### 2.4.1. First Evaluation

During the initial evaluation phase (1 week in April 2023), the researchers (MT and TY) implemented a comprehensive scoring system to rate each item on the 9-point scale (1 = “not essential” to 9 = “essential”) [15]. Each panelist independently reviewed the competencies and assigned scores based on their understanding of how necessary each item was for the care competencies of community citizens. The 10 panelists emailed the completed spreadsheets to the researchers after entering their scores.

#### 2.4.2. Panel Meeting

Panel meetings were held between the first and second evaluation rounds. During this meeting, each panelist was encouraged to discuss their reasoning behind the scores they assigned. Based on the RAND/UCLA method, we selected items with a median score of ≥7, for which at least three panelists scored between 1 and 6, or those with a median score of ≤3, for which at least three panelists scored between 4 and 9 [15]. Items that were clearly deemed appropriate for deletion were eliminated at the panel meeting if all panelists approved. This dialog fostered a collaborative environment where the panelists reached a consensus on the perceived value of each care competency item, ensuring that the final assessments reflected a collective agreement on their importance in the context of the care competencies of community members who are non-healthcare professionals. The median scores and number of panel members assigning ratings from 1 to 9 were calculated using Microsoft Excel.

#### 2.4.3. Second Evaluation

The 10 panelists repeated the scoring process for the second evaluation using the criteria established in the first evaluation. Each item was reevaluated, and the median score for each competency was calculated to gauge its overall importance. Items that received a median score of ≥7, for which two or fewer panelists scored between 1 and 6, indicating they were deemed essential, were retained. This rigorous evaluation process ensured that only the most essential care competencies were included in the final framework.

#### 2.4.4. Final Version of the Competencies

**Figure A1:**
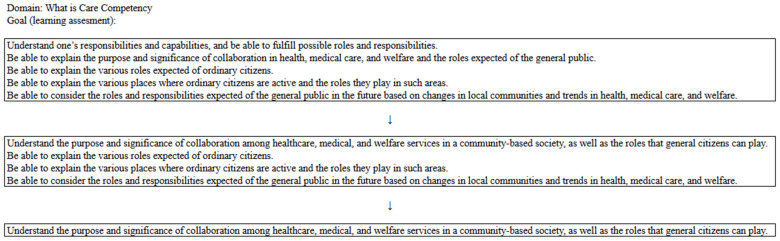
Process for merging similar items.

The final refinement of the care competency list was conducted after the modified Delphi study’s second evaluation round. This step aimed to clarify the items on the list by allowing panelists to discuss merging similar items with others identified in the second evaluation, as illustrated in Appendix A (Figure A1). The refinement process began with 227 essential items, some of which were then merged to ensure clarity regarding the elements included in the concept of care competencies. Ultimately, this refinement resulted in a final list of 151 competency items, which received unanimous approval from all panelists. Based on this procedure, the researchers aimed to establish a comprehensive list of care competencies that could inform educational programs for community members.

## 3. Results

### 3.1. Defining Care Competencies

We began by closely examining current health and care concepts to identify essential terms to include in our definition of care competency. Each researcher subsequently developed individual draft definitions of care competency based on these key terms. Through collaborative review and discussion, we reached a consensus to establish the final definition.

“Care competency” was defined as the “multidimensional ability to acquire and consciously utilize evidence-based knowledge and skills to sustain the life, health, and well-being of oneself, one’s family and friends, and people in the community. This ability encompasses a respectful attitude toward human dignity; an understanding of physical, psychological, and social health and well-being; and practical skills in care-related communication and actions.”

### 3.2. Delphi Surveys

**Figure 1:**
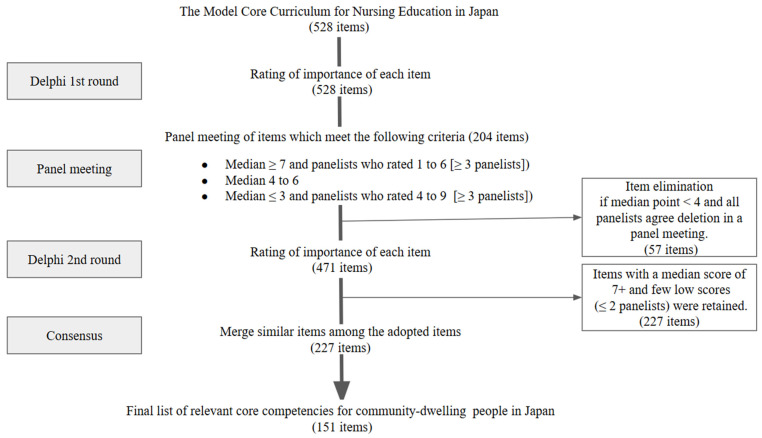
Flow diagram for the modified Delphi consensus process.

Figure 1 shows the flow diagram for the modified Delphi consensus process.

#### 3.2.1. Panelists

All 10 invited panel members (9 women, 1 man) participated in the two Delphi rounds. Each panelist held registered nurse qualifications, with an average of 12.8 years of experience as researchers.

#### 3.2.2. First Evaluation

The first evaluation comprised 528 items. None of the items had a median of 1–3; 79 had a median of 4–6, and 415 had a median of 7–9, of which 128 had at least three panelists with a median of 1–6. Among the 528 items, 204 met the panel-meeting criteria.

#### 3.2.3. Panel Meeting

The panelists shared how they scored the items. For example, for “Ability to judge the effects and side effects of treatments such as pharmacotherapy,” they commented, “I rated it lower since I thought the verb ‘judge’ might be beyond the ability expected of non-professional people”, and “I rated it lower; the phase of being able to judge is quite advanced.” The panel discussion revealed that during the evaluation, the influence of the verbs in the items overshadowed their actual content. Therefore, we removed the verbs from each item and revised them to focus on evaluating their content for the second scoring round. After the panel meeting, the panelists deleted 57 items from the second evaluation. These items had median points < 4, and their deletion was unanimously agreed upon during a panel meeting.

#### 3.2.4. Second Evaluation

The Second Evaluation began with 471 items. In total, 227 items were deemed essential (median of 7–9) with two or fewer disagreements (scored 1–3 by each panelist) for inclusion in the final framework.

#### 3.2.5. Final Version of the Competencies

**Figure A2:**
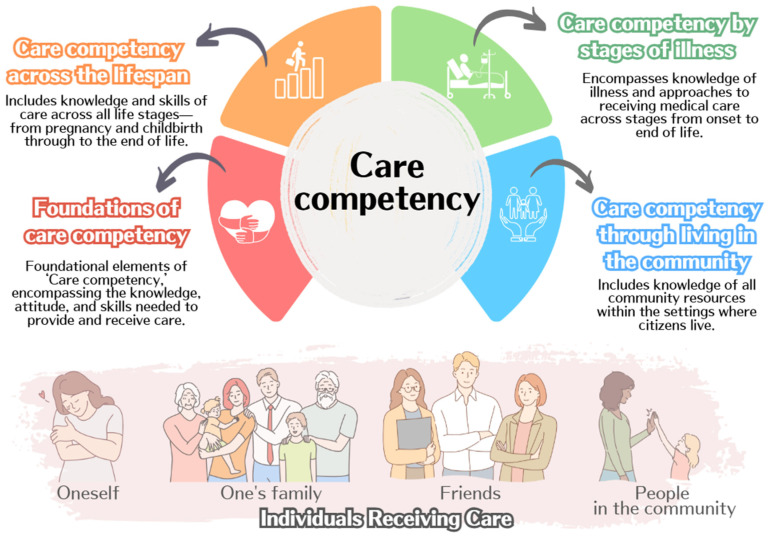
Visual diagram of the conceptual framework.

Since we planned to create educational programs, the 227 items were refined by merging similar items to clarify the elements included in the concept of care competencies. This resulted in a final list of four themes encompassing 23 domains and 151 items that received consensus from all panelists. Table 1 and Appendix B (Figure A2, Table A1 and Table A2) list the care competencies and core components.

The four themes were: foundations of care competency, care competency across the lifespan, care competency by stages of illness, and care competency through living in the community. The first, foundations of care competency, includes 76 items covering essential knowledge, attitudes, and skills such as health basics (nutrition, sleep, exercise), care techniques, ethics, communication, and medical safety. The second, care competency across the lifespan, consists of 31 items addressing care from pregnancy through aging and death, with domains like sexuality and life, family, children, adults, older adults, and advanced care planning. The third theme, care competency by stages of illness, has 18 items focusing on disease progression from onset to terminal phase, including mental healthcare and acute, recovery, chronic, and end-of-life care. Lastly, care competency through living in the community comprises 26 items related to social resources and systems, including support from schools and agencies, as well as disaster preparedness and care during emergencies.

## 4. Discussion

The aim of this study was to define the care competencies required for community citizens and clarify their components based on expert consensus using the Delphi method. This process resulted in a comprehensive list of 151 competency items, which were organized into four categories as follows: foundations of care competency, care competency across the lifespan, care competency by stages of illness, and care competency through community living. This framework provides a robust theoretical basis for developing citizen-oriented educational programs aimed at enhancing community members’ abilities to provide and receive care, thereby fostering a community-based, inclusive society [1]. Given the global challenge of rapidly aging societies, Japan, which is developing universal frameworks such as care competency, holds significant implications for global community health policy [1,2].

### 4.1. Concept Distinction and Boundaries

We defined “care competency” as the complex ability to acquire and utilize knowledge and skills, based on evidence and intentional choices, to maintain the well-being of oneself, loved ones, and people in the community. This concept explicitly integrates knowledge, skills, and attitudes, justifying its creation as a necessary framework for community-level care by non-professionals. Existing frameworks, such as health literacy [5,6,7] and community literacy [8], primarily focus on understanding and utilizing health-related information. However, these concepts, stemming from the literal meaning of “literacy,” lack the practical dimensions of caregiving, such as empathic attitudes, role readiness, and contextual decision-making, which are crucial for non-professionals. Care competency fills this critical gap by integrating the essential knowledge, skills, and attitudes required for active participation in caregiving.

The consensus process, especially the panel meeting discussions, provided valuable insight into the boundaries between professional and non-professional expectations. During the first evaluation, complex items, such as the “ability to judge the effects and side effects of treatments such as pharmacotherapy,” were frequently rated lower. Panelists commented that verbs such as “judge” might be beyond the ability expected of non-professionals, indicating that the ability to judge is quite advanced. The subsequent removal of such complex items and revision of language to focus on content clarified that care competency centers on foundational knowledge and practical support, rather than diagnostic or clinical judgment. This distinction is critical for defining appropriate roles for citizens in community care.

### 4.2. Strengths and Practical Application

The comprehensive list of 151 items, developed by adapting the rigorous Model Core Curriculum for Nursing Education in Japan [17], represents a pioneering effort to define generalizable care competencies beyond disease- or target-specific training programs. This framework provides a robust theoretical basis that can be leveraged to create educational programs.

However, we should address the practicality and feasibility of implementation given the volume and scope of the 151 items, and the fact that the target population is “all citizens.” A uniform program structure is unlikely to be realistic due to varying learning burdens, motivation, and life needs. Therefore, future implementation should adopt a flexible, modular, and phased educational approach. Examples of practical delivery formats include online learning, community-based workshops, microlearning resources, and e-learning. Implementation should also be integrated into existing societal structures using concrete examples, such as incorporating these competencies into school curricula, community volunteer programs, local emergency management training, or introductory modules focusing on concrete life challenges, including caring for older adults, parenting, or disaster response. This approach is expected to support the practical application of the work and encourage engagement from key stakeholders, including local governments, educators, and non-profit organizations.

### 4.3. Comparison with International Initiatives

The Social Determinants of Health (SDoH)—non-medical factors such as sociocultural, political, and economic environments—are widely recognized as fundamental drivers of health disparities [19,20]. Addressing these systemic inequities requires a comprehensive approach that extends beyond clinical care and engages broader social structures. The Care Competency Framework proposed in this study responds to this imperative by integrating both practical and systemic knowledge essential for navigating health-related challenges. In particular, the theme of “care competency through living in the community” highlights the importance of utilizing social resources, including welfare and social security systems, to empower individuals in overcoming barriers to care and promoting health equity. By positioning care as a shared civic responsibility, the framework aligns with global efforts to address SDoH through inclusive, community-based strategies.

Compared to existing international initiatives, the Care Competency Framework offers a distinct and more universal orientation. Programs such as the Community Health Worker education initiatives in the United States [21,22,23] and the Health (Community) Champions in the United Kingdom [24,25] train selected individuals—typically without formal medical qualifications—to provide basic health support and promote health awareness within their communities. While these models have demonstrated effectiveness in reaching underserved populations and fostering trust, they remain limited by role specificity, sustainability challenges, and uneven implementation. In contrast, the Care Competency Framework defines essential care-related skills for all citizens across the lifespan, encompassing both caregiving and care-receiving roles. This universality enables the development of tailored programs that reflect diverse life stages and social contexts. By offering a scalable and adaptable model, the framework helps to democratize care. It supports the advancement of inclusive, equity-oriented practices responsive to local and global SDoH conditions.

### 4.4. Limitations and Future Research Directions

Despite these strengths, our study has some limitations. First, regarding the panel composition, the care competencies were developed exclusively by a relatively small and homogeneous group of 10 nursing researchers, meaning the final competency list may reflect the priorities of this specific discipline. Although all panelists held registered nurse qualifications and had prior working experience in clinical or administrative settings, we did not detail the extent of their current clinical experience and engagement in frontline community-based practice.

Second, the practical applicability and feasibility of these 151 competencies for citizens is yet to be validated. Therefore, future studies should involve diverse stakeholders, including multidisciplinary practitioners, frontline professionals (e.g., visiting nurses, staff from community comprehensive support centers, or municipal health officials), informal caregivers, and community residents. Incorporating feedback from professionals who work directly with community members is essential to enhance the practical applicability and feasibility of implementation. Third, the large number of care competency items is a limitation. Statistical methods such as factor analysis could be beneficial to enhance the efficiency and practicality of the final list.

Finally, this study focused solely on clarifying the concept and creating the list. Future research should focus on the empirical validation of this framework with citizens and informal caregivers (including individuals with caregiving experience). In addition, this framework’s applicability should be tested in different cultural contexts beyond Japan to confirm its global relevance. Future steps include developing scales to measure care competency cultivation and the learning effects of educational programs. Moreover, continuous implementation, participant feedback, and improvement are necessary to ensure the list remains comprehensive and relevant.

## 5. Conclusions

This study defined care competency and established a comprehensive 151-item framework for community citizens. Notably, this novel framework provides a practical basis for developing globally applicable educational programs for rapidly aging societies. Aimed at non-professionals, the categorized competencies can transform existing fragmented training into tailored, comprehensive community care education. Future research, including validation with diverse stakeholders and developing measurement scales, is essential to refine the model’s practical applicability.

## Figures and Tables

**Table 1:** Overview of care competencies.

	Themes	Domains
Care competency	Foundations of care competency (76 items)	What care competency is Basics of health Basics of care Engaging with “health” Engaging with “care” Care techniques Ethics Understanding of research Medical safety
	Care competency across the lifespan (31 items)	Sexuality and life Family Children Adults Older adults Advance care planning
	Care competency by stages of illness (18 items)	Fundamentals of illness Mental health Acute phase Recovery phase Chronic phase Terminal phase
	Care competency through living in the community (26 items)	Community systems supporting care Disaster preparedness

## Data Availability

The original contributions presented in this study are included in the article. Further inquiries can be directed to the corresponding author.

## Abbreviations

The following abbreviations are used in this manuscript:

UCLA	University of California, Los Angeles
SDoH	Social Determinants of Health

## Appendix B

**Table A1:** Outline of care competencies.

	Themes	Domains	
Care competency	Foundations of care competency (76 items)	What care competency is Basics of health Basics of care Engaging with “health” Engaging with “care” Care techniques Ethics Understanding of Research Medical safety	This theme forms the foundation of care competencies, encompassing the knowledge, attitudes, and care skills required to provide and receive care. For example, the basics of health include the definition of health, maintaining a healthy environment and lifestyle, food, nutrition, elimination, exercise, and sleep, among others. The basics of care encompass the definition of care, thought processes and techniques involved in providing care, and essential communication skills required for effective care.
	Care competency across the lifespan (31 items)	Sexuality and life Family Children Adults Older adults Advance care planning	Pregnancy; childbirth; and each process of birth, aging, and death, including knowledge, attitudes, and care skills. For example, sexuality and life include the various sexes and the perinatal period (pregnancy through postpartum care). Family encompasses the process of family formation and its associated functions.
	Care competency by stages of illness (18 items)	Fundamentals of illness Mental health Acute phase Recovery phase Chronic phase Terminal phase	This includes knowledge and preparedness regarding the patient’s disease transition process from the onset of illness to the end of life. For example, the fundamentals of illness encompass the physical and mental reactions to the illness, alongside its primary symptoms. The acute phase includes the physical and psychological characteristics of the individual in critical condition, alongside the psychological and emotional anxiety of their family.
	Care competency through living in the community (26 items)	Community systems supporting care Disaster preparedness	This theme encompasses knowledge and care skills related to social resources and community structures where citizens reside. For example, community systems supporting care encompass the characteristics of the school setting, government agencies, and community features. Disaster preparedness encompasses healthcare in the community during a disaster and the care required at each stage of disaster relief efforts.

**Table A2:** Lists of care competencies and core components.

Themes	Domains	Goals (Learning Assessment)
Foundations of care competency	What care competency is	Understand what care competency entails.
		Understand the purpose and significance of collaboration among healthcare, medical, and welfare services in a community-based society, as well as the roles that general citizens can play.
	Basics of health	Be able to consider the meaning of health from one’s own perspective.
		Understand basic human needs.
		Understand the changes in daily activities, emotions, communication, and social roles that occur with human growth and development.
		Recognize that a healthy lifestyle varies from person to person.
		Understand the relationship between health and factors such as the environment, nutrition/diet, excretion, activity, and rest.
		Understand the influence of genetics and the environment (sociocultural, physio-bio-chemical, political, and economic environments) on health.
		Understand the structure of dietary habits, factors affecting eating behaviors, and the significance of food for health.
		Understand nutrition and energy metabolism.
		Understand the structure and function of the digestive tract and glands related to nutrition and excretion.
		Recognize the significance of excretion for health.
		Understand the balance and rhythm (biological rhythms, exercise habits, sleep patterns, work/activity, and leisure) necessary for maintaining health.
		Understand and respect the diversity of people’s values and social backgrounds in relation to a healthy and fulfilling life.
		Understand the relationship between addictions (such as smoking, drinking, and gambling) and health.
		Recognize the importance of considering individual lifestyles (such as daily habits, purpose in life, and religious practices) from a health and care perspective, considering diverse cultural backgrounds.
	Basics of care	Be able to think about what care is and its purpose.
		Understand that care aims to ensure the safety, comfort, and independence of the care recipient.
		Recognize the importance of supporting the care recipient within a care team centered on them.
	Engaging with “health”	Recognize that one’s health is one’s own responsibility and possess the ability to engage proactively with medical care (understand how to seek a second opinion, how to respond when referred to a clinic in an outpatient setting, and how to approach medical consultations).
		Be able to critically evaluate various types of information related to health and daily life, as well as select and utilize relevant information.
		Be able to manage one’s health condition and take action to seek support from others when necessary.
		Be able to consider ways to prevent illnesses and disabilities.
		Understand awareness-raising activities aimed at promoting an accurate understanding of physical and mental health disorders.
		Understand the health checkup systems necessary for early detection, diagnosis, and treatment of physical and mental health disorders, as well as how to utilize them.
		Recognize symptoms and situations that require urgent medical attention.
		Be knowledgeable about hospital admission and discharge preparations appropriate for different illnesses and disabilities.
		Be able to implement infection prevention measures.
	Engaging with “care”	Develop an active attitude toward learning about care.
		Understand that communication is crucial to relationships related to care.
		Recognize one’s communication tendencies and engage with care recipients while being aware of personal challenges.
		Use various communication methods (such as verbal, non-verbal, written, and visual information) to build relationships tailored to the care recipient’s characteristics.
		Understand that providing care requires accurate knowledge, reliable skills, and an appropriate attitude.
		Recognize the importance of implementing care based on a plan, evaluating the outcomes, and improving future care.
		Show interest in the care recipient.
		Understand how to apply knowledge about care to support the care recipient in achieving better health.
		Recognize the need to gather diverse information to gain a holistic understanding of the care recipient.
		Be able to collect as much relevant information as possible based on the care recipient’s condition.
		Integrate collected information to consider the care recipient’s lifestyle and health status from multiple perspectives.
		Support care recipients in making informed decisions for maintaining and improving their health.
		Understand methods for evaluating whether the care provided was effective.
	Care techniques	Be able to adjust the care recipient’s posture to ensure they remain in a comfortable position.
		Be able to make adjustments that help the care recipient remain mentally calm.
		Be able to create an environment necessary for the care recipient to live safely and comfortably.
		Be able to support the care recipient in eating safely and comfortably.
		Be able to support the care recipient in excreting safely and comfortably.
		Be able to support the care recipient in engaging in daily activities and resting safely and comfortably.
		Be able to support the care recipient in maintaining cleanliness and appropriate clothing habits.
		Be able to support the care recipient in maintaining stable respiratory and circulatory conditions.
		Be able to perform necessary measurements (such as body temperature, SpO_2_, and blood pressure) to assess the physical and mental condition of the care recipient.
		Be able to support the care recipient in taking medication safely, including self-care.
		Be able to perform basic life support (such as using an automated external defibrillator) and first aid (emergency care).
	Ethics	Understand that fundamental human rights form the foundation of care practice within one’s responsibilities and capabilities.
		Maintain an attitude that respects life and human dignity.
		Understand the means and methods to protect people’s fundamental human rights in daily care (including the significance and necessity of informed consent and assent, confidentiality, and methods of protecting personal information).
		Recognize that care is an interaction between the care recipient and provider and that the rights of care providers must also be protected.
		Be able to uphold confidentiality and the protection of personal information.
	Understanding of research	Understand how research findings are applied to address social issues related to health.
		Recognize the necessity of ethical considerations in medical and care-related research.
	Medical safety	Understand that medical treatment carries both benefits and risks (such as complications and side effects).
		Understand the effects of drugs and radiation on health and daily life.
		Understand safe care practices and methods for preventing adverse events related to care (such as falls, pressure ulcers, and medication errors).
		Recognize the safety measures required in medical, caregiving, and welfare services, as well as the role of care recipients in ensuring their safety.
		Understand the importance of systems for the safe management of pharmaceuticals and medical devices, as well as the significance of building a safe medical environment.
		Recognize the importance of reflecting on accidents and near-miss incidents that occur at home.
Care competency across the lifespan	Sexuality and life	Understand the health issues specific to each stage of a woman’s life cycle and the necessary care associated with them.
		Understand the diversity of sexuality.
		Recognize social issues related to people’s sexual and reproductive health and rights.
		Understand maternal and child health, as well as postpartum care, childcare support, and maternal and child health systems.
		Understand care that supports parent–child bonding and family development during the perinatal period.
		Understand sexuality and reproduction in daily life.
		Recognize the physical, psychological, and social characteristics, as well as physiological changes during pregnancy, childbirth, postpartum, and the neonatal period.
		Be able to implement necessary support for health promotion during pregnancy, childbirth, postpartum, and the neonatal period.
	Family	Understand the developmental tasks of individuals and families.
		Recognize the process of childbirth and family development, as well as the functions of the family.
		Understand the self-care functions of families.
	Children	Recognize the importance of protecting children’s rights.
		Understand children’s growth and development, along with appropriate care methods for each stage.
		Be aware of issues related to the growth, development, and health of children and the care needs of children in hospitals, homes, schools, and other settings.
		Understand the impact of illness and hospitalization on children.
		Recognize child-specific care techniques, including childcare-related support and emergency response.
		Be able to implement necessary support for children’s self-care acquisition and overall growth and development.
	Adults	Be able to consider health issues in adulthood from physical, psychological, and social perspectives.
		Be able to consider the balance between work and family life when managing necessary medical care and self-care.
	Older adults	Be able to comprehensively consider the situation of older adults receiving care, considering age-specific physical, psychological, and social changes, as well as individual lifestyles, values, and spirituality.
		Understand health risks specific to older adults (such as falls, pain, delirium, cognitive decline, depression, malnutrition, swallowing disorders, and pressure ulcers) and prevention methods.
		Understand ways to support older adults and their families in maintaining individuality while maximizing their abilities, based on their self-care capabilities.
		Recognize how older adults can live authentically by accessing support from various professionals and institutions according to different health levels.
		Understand the characteristics of individuals with dementia and the appropriate care for them.
		Recognize the types and characteristics of older adult abuse and the roles that citizens can play in addressing them.
		Be able to consider care that supports the dignity and quality of life of older adults.
		Understand frailty, sarcopenia, locomotive syndrome, and their prevention methods.
	Advance care planning	Be able to consider end-of-life care based on the values of individuals in the final stages of life, including their views on life and death.
		Recognize the importance of collaborating with relevant institutions and professionals to support individuals in living their final stage of life in a way that aligns with their wishes.
		Understand the characteristics of the decision-making process for individuals in the final stage of life and the methods to support their decisions.
		Recognize the need for personalized care that considers values, family, and social backgrounds at the end of life.
Care competency by stages of illness	Fundamentals of illness	Understand the characteristics and treatment progress of different stages of diseases.
		Recognize major symptoms (such as consciousness disorders, seizures, hematemesis/hemoptysis, chest pain, oliguria/anuria/frequent urination, and pain, including chronic pain) and their management.
		Understand the physical and psychological responses of individuals to illness.
		Recognize how illness and disabilities impact family life and relationships among family members.
		Understand palliative care from a holistic perspective for individuals with illnesses.
		Be able to consider the distress and anxiety experienced by individuals with symptoms.
	Mental health	Be able to consider mental health issues.
		Understand the relationship between stress and health, as well as the causes of stress.
		Be able to identify early signs of mental illness in oneself and others and connect them to appropriate support.
		Recognize developmental challenges and psychological crises in different life cycle stages.
		Understand methods to improve mental health in settings such as homes, schools, and workplaces.
		Recognize the importance of supporting perinatal mothers and families to maintain mental health and promote the healthy psychological development of children.
		Understand developmental disorders and ways to provide appropriate environmental support.
		Be aware of available support resources for suicide prevention that can be accessed by individuals at risk and their families.
		Understand the support available for individuals with addiction and their families.
		Understand the stages of recovery and the corresponding care required at each stage, from hospitalization to discharge support, for individuals with mental illness.
		Understand the need for and methods of collaboration with relevant parties to provide community living support for individuals with mental illness.
	Acute phase	Understand the physical, psychological, and social characteristics of individuals in the acute or severe phase.
		Understand the psychology and anxiety of individuals in the acute or severe phase, and those of their families.
	Recovery phase	Understand the situation of families supporting individuals recovering from illness or disability.
		Understand the concept of normalization.
		Understand functional disabilities (such as physical, intellectual, higher cognitive, mental, and developmental) and the concept of rehabilitation.
		Understand the social resources available for individuals in the recovery phase to live with their disabilities.
	Chronic phase	Understand the foundational principles of support for individuals with chronic illnesses.
		Be able to assess one’s own or a family member’s self-care status and challenges based on awareness of illness or disability, self-management status, and test results.
		Understand the causes of sudden deterioration of illness and the need for prevention.
	Terminal phase	Be able to think about the concept of death and the meaning of death, as well as end-of-life care for individuals and their families.
		Understand the physical changes in individuals at the final stage of life.
		Understand grief counseling for individuals who have lost a close person (such as family, friends, and acquaintances).
Care competency through living in the community	Community systems supporting care	Understand the concept of community-based integrated care.
		Understand self-help, mutual aid, cooperative support, and public assistance in the Integrated Community Care System.
		Understand the meaning of dependence on others and independence in society, and how this balance impacts daily life.
		Understand the necessity of health support in familiar communities for individuals at various life cycle stages and health levels.
		Understand the necessity of considering the health conditions and care needs of all individuals living in the community.
		Understand the types of social security systems (such as social insurance, public assistance, and social welfare).
		Understand the types of social insurance (health insurance, pension insurance, workers’ compensation insurance, employment insurance, and long-term care insurance).
		Understand the roles of various health, medical, and welfare institutions and how to utilize them to achieve the desired way of living.
		Understand the social resources available for community residents, home-care patients, and their families.
		Understand the types and functions of medical facilities providing healthcare.
		Understand the characteristics of home care institutions such as visiting nursing offices, nurse-led comprehensive community care, community comprehensive support centers, and support centers for children and families.
		Understand how medical and long-term care services are provided in care insurance-related service institutions.
		Understand the characteristics of welfare facilities (residential and daycare) for mothers and children, older adults, and individuals with physical and mental disabilities, among others.
		Understand how occupational health services are provided in companies.
		Understand how health services are provided in schools.
		Understand how health services are provided in national and local government administrations.
		Be able to gather information about regional health, medical, and welfare systems.
		Understand the local healthcare system and the division of roles among various institutions in one’s community.
	Disaster preparedness	Understand the impact of disasters on health and daily life.
		Understand disaster types, disaster cycles, regional disaster prevention plans, and support systems.
		Understand that different stages of medical relief activities exist during disasters and the corresponding care required at each stage.
		Understand assistance for daily life (e.g., meals, excretion, sleep, hygiene, and environmental maintenance) and physical/mental health management at living facilities during disasters (rescue stations, evacuation centers, welfare evacuation centers, temporary housing, and damaged medical facilities), including self-care.
		Understand the care needs of vulnerable individuals and those requiring evacuation assistance during disasters.
		Understand the necessity for and methods of cooperation and collaboration with disaster-affected communities and multi-professional teams for care.
		Understand the occurrence and risks of secondary disasters.
		Understand stress and mental healthcare for disaster victims and relief workers, including self-care.
